# ARG2, MAP4K5 and TSTA3 as Diagnostic Markers of Steroid-Induced Osteonecrosis of the Femoral Head and Their Correlation With Immune Infiltration

**DOI:** 10.3389/fgene.2021.691465

**Published:** 2021-07-26

**Authors:** Rongguo Yu, Jiayu Zhang, Youguang Zhuo, Xu Hong, Jie Ye, Susu Tang, Nannan Liu, Yiyuan Zhang

**Affiliations:** ^1^Department of Orthopaedics, Fuzhou Second Affiliated Hospital, Xiamen University, Xiamen, China; ^2^Fuzhou Second Affiliated Hospital, Xiamen University, Xiamen, China; ^3^School of Clinical Medicine, Yunnan University of Traditional Chinese Medicine, Kunming, China; ^4^Fuzhou Second Hospital Affiliated to Xiamen University, Fujian, China

**Keywords:** steroid-induced osteonecrosis of the femoral head, immune cells, diagnostic marker, gene expression omnibus, biomarker

## Abstract

**Background:**

The diagnosis for steroid-induced osteonecrosis of the femoral head (SONFH) is hard to achieve at the early stage, which results in patients receiving ineffective treatment options and a poor prognosis for most cases. The present study aimed to find potential diagnostic markers of SONFH and analyze the effect exerted by infiltration of immune cells in this pathology.

**Materials and Methods:**

R software was adopted for identifying differentially expressed genes (DEGs) and conducting functional investigation based on the microarray dataset. Then we combined SVM-RFE, WGCNA, LASSO logistic regression, and random forest (RF) algorithms for screening the diagnostic markers of SONFH and further verification by qRT-PCR. The diagnostic values were assessed through receiver operating characteristic (ROC) curves. CIBERSORT was then adopted for assessing the infiltration of immune cells and the relationship of infiltration-related immune cells and diagnostic markers.

**Results:**

We identified 383 DEGs overall. This study found ARG2, MAP4K5, and TSTA3 (AUC = 0.980) to be diagnostic markers of SONFH. The results of qRT-PCR showed a statistically significant difference in all markers. Analysis of infiltration of immune cells indicated that neutrophils, activated dendritic cells and memory B cells were likely to show the relationship with SONFH occurrence and progress. Additionally, all diagnostic markers had different degrees of correlation with T cell follicular helper, neutrophils, memory B cells, and activated dendritic cells.

**Conclusion:**

ARG2, MAP4K5, and TSTA3 are potential diagnostic genes for SONFH, and infiltration of immune cells may critically impact SONFH occurrence and progression.

## Introduction

SONFH refers to a general and disabling orthopedic disease, particularly after the 2003 severe acute respiratory syndrome (SARS) (Guo et al., 2014). The incidence of SONFH has been rising in recent years, with approximately 20,000–30,000 novel cases diagnosed in the US each year (Lieberman et al., 2003). Each year, the morbidity rate reaches 1.91/100,000 in Japan (Ikeuchi et al., 2015). High doses or long-term use of steroid hormones have been suggested to be correlated with SONFH (Han et al., 2010; Zhao et al., 2016). During the continuing novel coronavirus pandemic since 2019 (COVID-19), over 70 million cumulative cases have been reported with COVID-19, as of December 13, 2020 (Covid-19 National Incident Room Surveillance Team, 2021), and the incidence of SONFH is expected to rise.

Most cases of SONFH present with femoral head collapse within 1–3 years of diagnosis (Fu et al., 2019) and will require artificial joint replacement. Although the quality of life of patients with SONFH has been improved through surgical techniques and prosthetics, many cases may still suffer from psychology- and economic-related burdens after receiving total hip arthroplasty (Tripathy et al., 2015). Effective treatment requires early diagnosis. Joint imaging techniques are routinely undertaken in the diagnosis of SONFH, but these do not enable a precise early diagnosis (Ando et al., 2021). Since we lack a reliable method of early diagnosis, the progress of the disease in most cases results in ineffective treatment options and poor prognosis. It is, therefore, crucial to explore the biological markers that could make an early diagnosis possible.

Over the past few years, studies have increasingly suggested that infiltration of immune cells significantly impacts the occurrence and development of femoral head necrosis. Chronic inflammatory reactions occurred in and around the necrotic area of the femoral head, and the changes of T-lymphocyte subsets may affect various inflammatory reactions (Tao et al., 2017). The neutrophil percentages and mononuclear macrophage content led to a higher incidence of femoral head necrosis (Jiang et al., 2019). Additionally, from the perspective of the immune system, the evaluation of the degree of infiltration of immune cells and the determination of the differences in the components of infiltration-related immune cells are of high significance for illustrating the molecular pathological mechanism of SONFH and developing novel immunotherapeutic targets. As a gene-based deconvolution algorithm, CIBERSORT is an analysis tool to evaluate the degree of immune cell infiltration from samples (Newman et al., 2015). The analysis of immune cell infiltration has been widely used in a variety of diseases (e.g., osteoarthritis (Deng et al., 2020), cervical cancer (Yang et al., 2021), lung adenocarcinoma (Ren et al., 2020). However, no studies have been conducted to analyze the infiltration of immune cells in SONFH and assess its value using CIBERSORT.

In the present study, machine learning algorithms and multiple bioinformatic approaches were initially used in combination to screen and determine the diagnostic markers of SONFH based on the microarray dataset, as downloaded from the Gene Expression Omnibus (GEO) database. Furthermore, this study initially employed CIBERSORT to assess the difference of immune infiltration between normal tissues and SONFH tissues in 22 immune cell subsets. To gain more insights into the underlying molecular immune mechanism during the development of SONFH, this study explored the relationship between infiltration-related immune cells and diagnostic markers.

## Materials and Methods

### Data Collection

The first search for datasets was conducted here in the GEO database^1^ based on ‘‘femoral head necrosis’’ + ‘‘steroid,’’ followed by selecting ‘‘Homo sapiens’’ + ‘‘Expression profiling by array.’’ The screening standards were as follows: The microarray datasets were gene expression profiles with genome-wide of Human peripheral serum; the microarray datasets contain normal and SONFH samples. All included samples were treated with high-dose and (or) long-term administration of steroid hormones. Finally, GSE123568^2^ was used for subsequent analysis of 30 SONFH samples (17 women, 13 men; ARCO I–II 10, stage III 10, stage IV 10; mean age: 23.07 ± 3.01 years) and 10 non-SONFH samples (3 women, 7 men; mean age 25.02 ± 2.87 years).

### Data Preprocessing and Identification of DEGs

First, the raw data used to do the background correction and normalization by robust multiarray averaging (RMA) with the “affy” package (Gautier et al., 2004). Next, the limma package was adopted to identify the DEGs between SONFH cases and non-SONFH cases in R software (Ritchie et al., 2015). *P*-values were adjusted using the Benjamini and Hochberg test, and *p* < 0.05 and | log2FC| > 1 were considered the cutoff criterion.

### Functional Correlation Analysis

The “clusterProfiler” and “ggplot2” package were employed for Gene Ontology (GO) and the Kyoto Encyclopedia of Genes and Genomes (KEGG) was used for enrichment analyses of DEGs (Yu et al., 2012). The “clusterProfiler” and “ggplot2” packages were also used to perform Gene set enrichment analysis (GSEA) on the gene expression matrix, *p* < 0.05 were considered significant enrichment.

### Screening and Verification of Diagnostic Markers

We used the least absolute shrinkage and selection operator (LASSO) logistic regression (Suykens and Vandewalle, 1999), support vector machine-recursive feature elimination (SVM-RFE) (Tibshirani, 1996), random forests (RF) (Breiman, 2001), and weighted gene co-expression network analysis (WGCNA) (Langfelder and Horvath, 2008) to perform feature selection to screen diagnostic markers for SONFH. The LASSO algorithm was applied with the “glmnet” package (Friedman et al., 2010). Next, SVM-RFE refers to a machine learning method by complying with a support vector machine, which was adopted for finding the optimal variables through the deletion of SVM-generated eigenvectors. An SVM module was built for further identifying the diagnostic significance of the mentioned biological markers in SONFH based on the “e1071” package (Huang et al., 2014). The random forest (RF) algorithm randomized the algorithm for reduction of a single decision tree overfitting and the promotion model accuracy based on numerous relevant decision trees from one training set (Gers and Schmidhuber, 2001). WGCNA is capable of integrating gene expressing state and trait information for identifying function-related channels and candidate biological markers (Presson et al., 2008). The genes from these four classification models algorithms were obtained and used for further analysis. For the in-depth tests of the diagnosis efficacy of ARG2, MAP4K5, and TSTA3, the diagnosis significance was assessed based on the investigation of receiver operating characteristic (ROC) curves (MedCalc software). The area under the curve (AUC) was a critical diagnosis-related index. A two-sided *p* < 0.05 showed statistical significance.

### Quantitative PCR Analysis

To further verify the prediction results, the present study adopted quantitative reverse transcriptase PCR (qRT-PCR) to examine the expression of the three diagnostic markers which were associated with aberrant expression in the serum of SONFH. We collected patients who had a medical history of treatments with high-dose or long-term hormones. All patients were enrolled from October 1, 2020, to March 1, 2021. A refrigerator at −80°C was used to store the collected serum samples.

The study was approved by the Ethical Committee of Fuzhou Second Hospital affiliated with Xiamen University. Each patient signed the written consent.

Total RNA was extracted from each sample using TRIzol reagent (TAKARA, Dalian, China). The cDNA reverse transcription kit (Applied Biosystems) was employed to reverse-transcribe RNA. Besides, the SYBR Green PCR kit (Qiagen, Germany) was used to amplify the resultant cDNA. GAPDH was then handled as an internal reference. Relative mRNA expression was calculated by the 2-ΔΔCt method. The mean of 2 continuous variables were compared by independent samples Student’s *t*-test or Mann–Whitney U test when appropriate. *P-*values < *0.05* showed statistical significance. Subsequently, a *t*-test was performed to calculate differences between groups. The primer sequences included GAPDH forward, CTCCTCTGACTTCAACAGCGACA, GAPDH reverse, GCT GTAGCCAAATTCGTTGTCAT; ARG2 forward, CGATGGA GGAGGTTAAAAGG, ARG2 reverse, CCCTGGATGTCAGA GAAAAG; MAP4K5 forward, CATTTATCTACGAGGCACCC, MAP4K5 reverse, CAGTGATCTCCTAAGAACCG; TSTA3 forward, CTCTCCAGTTTGGGTAAGTG, TSTA3 reverse, GGGTGATGGAATAAGACTCC.

### Evaluation of Infiltration of Immune Cells

The gene expression matrix information was uploaded into CIBERSORT (Newman et al., 2015). The samples with *p* < 0.05 received the filtering process and obtained the infiltration of the immune cells matrix. Then, we used the “ggplot2” package to perform PCA clustering analysis on the infiltration of immune cells matrix data for generating a 2D PCA clustering map. Principal component analysis (PCA) was a popular technique to be utilized as a form of multidimensional scaling (Jolliffe and Cadima, 2016). PCA could identify the underlying variables that explain the pattern of correlations within a set of observed variables and explore the latent structure of the variables in the data file according to two groups (normal and SONFH patients). For the visualization of the relationship of 22 types of infiltration-related immune cells, the “corrplot” package (Friendly, 2002) was employed for generating a correlation heatmap. For the visualization of the distinction in the infiltration of immune cells, the “ggplot2” package was employed for drawing violin diagrams. *P-*values < 0.05 showed statistical significance.

### Correlation Analysis Between Identified Genes and Infiltration-Related Immune Cells

The association of the identified gene biological markers with the levels of infiltration-related immune cells was explored using Spearman’s rank correlation analysis in R software. The resulting associations were visualized using the chart technique with the “ggplot2” package. *P-*values < 0.05 showed statistical significance.

## Results

### Data Preprocessing and DEGs Screening

**FIGURE 1:**
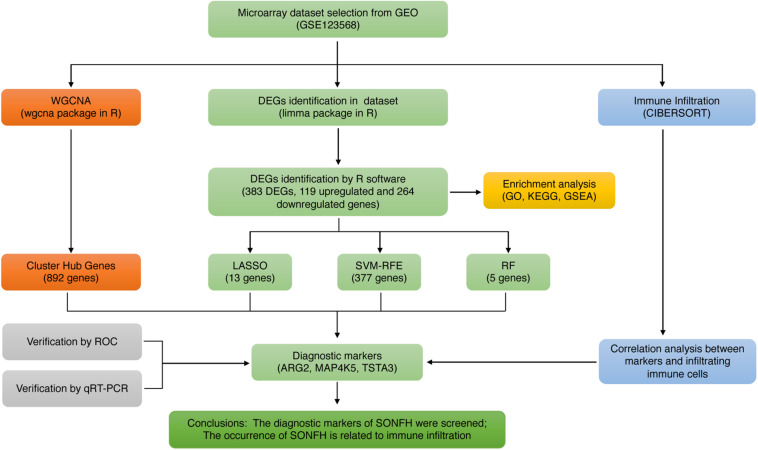
The flowchart of the analysis process.

**FIGURE 2:**
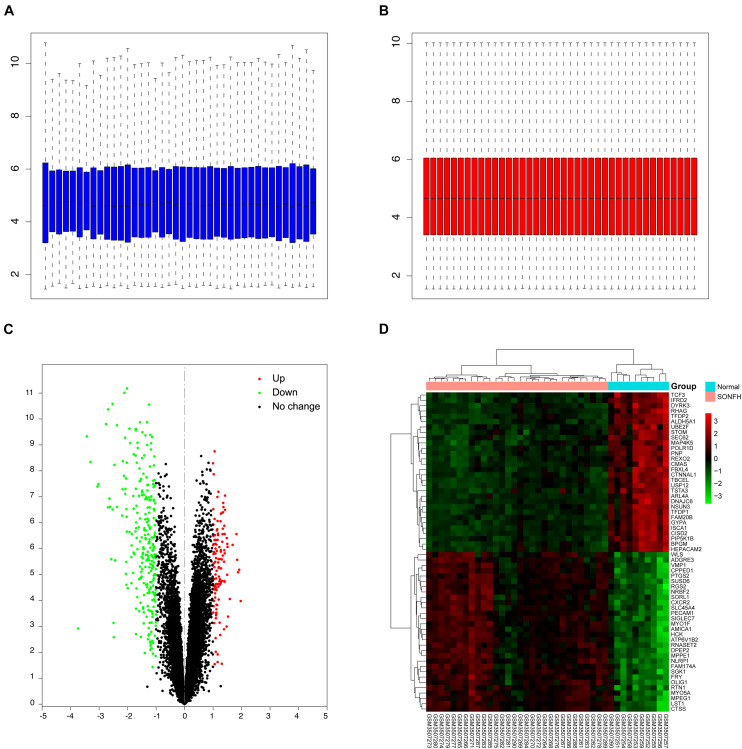
Data preprocessing and DEGs screening. **(A,B)** Box plots of the expression profiles: Before normalization (blue) and after normalization (red); **(C)** volcano map of DEGs; red represents up-regulated differential genes, black represents no significant difference genes, and green represents down-regulated differential genes; **(D)** heat map of top 30 up-regulated and down-regulated genes from all samples; red indicates higher gene expression and green indicates lower gene expression.

The workflow of this study is illustrated in Figure 1. Box plots before and after normalization of the raw data are shown in Figures 2A,B. After preprocessing, the present study adopted R software to extract a total of 383 DEGs from the gene expression matrix, including 119 up-regulated and 264 down-regulated genes (Supplementary List 1), as shown in the volcano map (Figures 2C,D).

### Functional Enrichment Analyses

**FIGURE 3:**
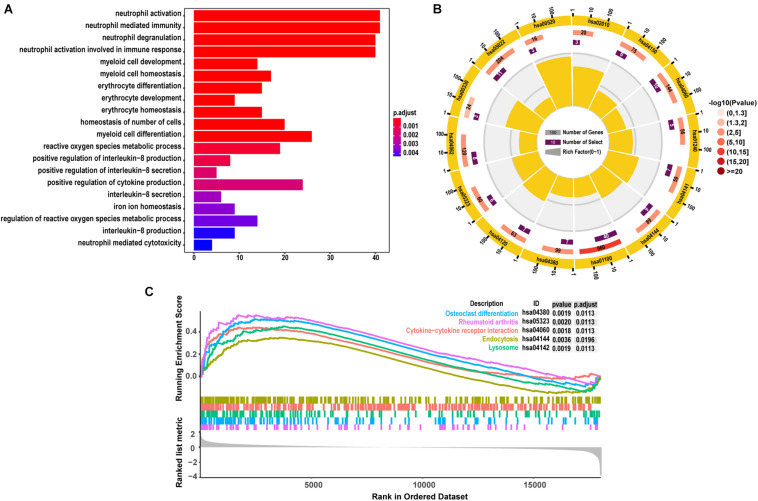
The results of functional enrichment analyses. **(A)** GO analyses results of DEGs; **(B)** pathway analyses results of DEGs; **(C)** the top 5 signal pathways were most related to the pathology of SONFH from the of results GSEA.

As demonstrated from the results of GO analysis, DEGs were mainly related to neutrophil activation, neutrophil mediated immunity, neutrophil degranulation, and neutrophil activation involved in immune response (Figure 3A). KEGG enriched by DEGs mainly included 14 pathway analyses (Figure 3B). GSEA results showed that the enriched pathways mainly involved Osteoclast differentiation, Rheumatoid arthritis, Cytokine-cytokine receptor interaction, Endocytosis, and Lysosome (Figure 3C), and all the results also were included in the results of KEGG.

### Screening and Verification of Diagnostic Markers

**FIGURE 4:**
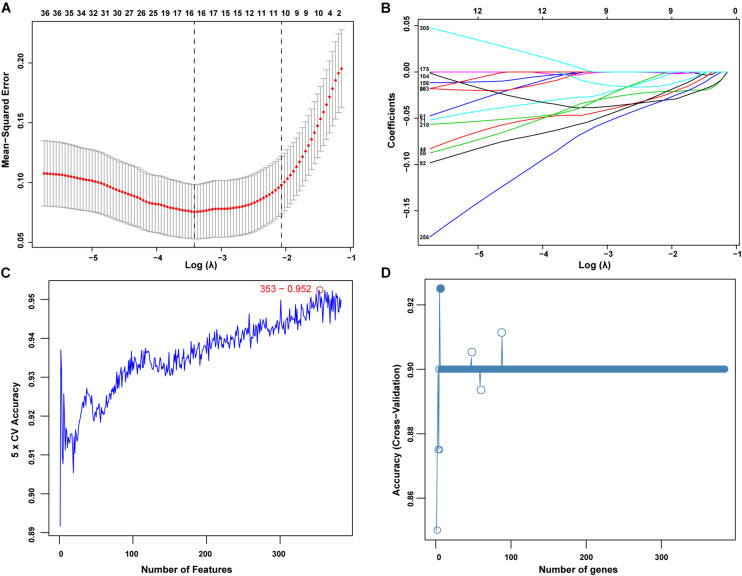
Screening of diagnostic markers via the comprehensive strategy. **(A,B)** Least absolute shrinkage and selection operator (LASSO) logistic regression algorithm to screen diagnostic markers; Different colors represent different genes; **(C,D)** based on support vector machine recursive feature elimination (SVM-RFE) and random forest (RF) algorithm to screen biomarkers.

**FIGURE 5:**
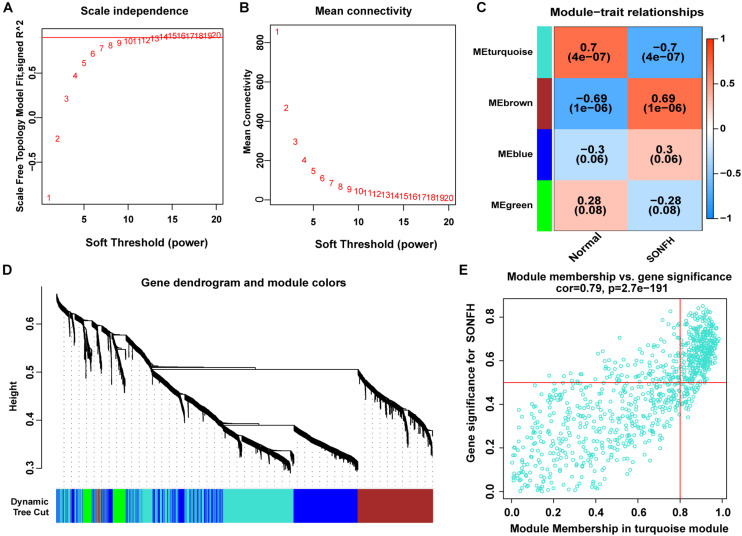
WGCNA revealed gene co-expression networks in the whole peripheral blood of 40 SONFH patients. **(A,B)** Analysis of the scale-free fit index (left) and the mean connectivity (right) for various soft-thresholding powers. **(C)** Heatmap between the correlation between modules and SONFH (Each cell contained the correlation coefficient and corresponding *P*-value). **(D)** Clustering dendrogram of differentially expressed genes related to SONFH. **(E)** The gene significance for SONFH in the turquoise module (One dot represents one gene in the turquoise module).

The present study adopted LASSO logistic regression algorithm to identify 13 genes from DEGs as diagnostic markers for SONFH (Figures 4A,B). 377 genes were identified from DEGs as diagnostic markers using the SVM-RFE algorithm (Figure 4C). Besides, 5 genes were identified from DEGs using the RF model as diagnostic markers (Figure 4D). When 0.9 was used as the correlation coefficient threshold, the soft-thresholding power was selected as 15. Based on WGCNA analysis, 15 co-expression modules were built (Figures 5A,B). As indicated from investigations of module-trait correlations, multiple modules displayed the relationship to SONFH (Figure 5C), and the turquoise module was the most significant example. Figure 5D shows the significance of the mentioned genes in the turquoise module for SONFH, with a total of 892 genes included (Figure 5E).

**FIGURE 6:**
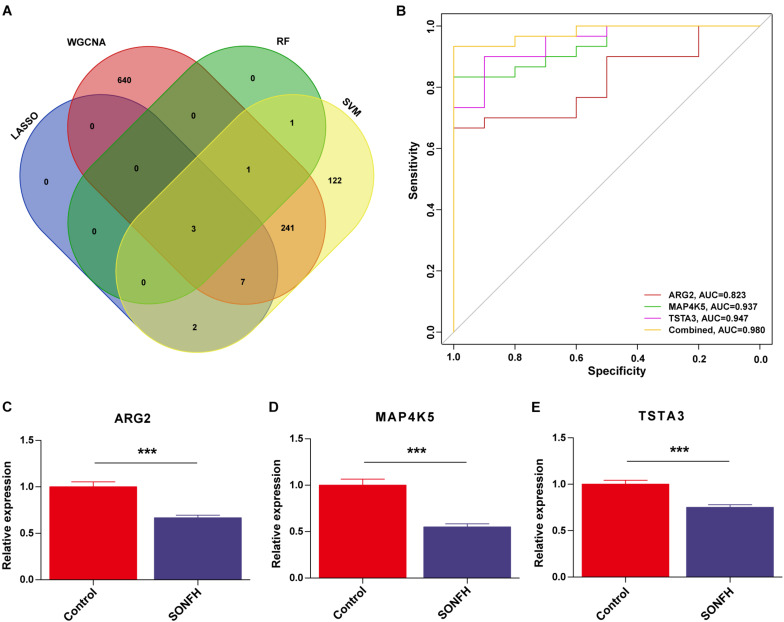
**(A)** Venn diagram showed the intersection of diagnostic markers obtained by the four algorithms. **(B)** The ROC curve of the diagnostic efficacy verification after fitting three diagnostic markers to one variable. **(C–E)** Validation of the expressions of potential diagnostic markers via qRT-PCR. *P < 0.05; **P < 0.01, ***P < 0.001.

The gene markers obtained by the four algorithms were overlapping. Lastly, three diagnostic related genes were obtained (Figure 6A). The ROC curves of ARG2, MAP4K5, and TSTA3 revealed the probability of them as valuable biological markers with AUCs of 0.823, 0.937, and 0.947, respectively (Figure 6B), indicating that the three diagnostic markers had a high diagnostic value. qRT-PCR was used to measure the miR expressions of diagnostic markers. On the whole, 24 serum samples with SONFH 16 serum samples with non-SONFH were collected at Fuzhou Second Hospital Affiliated with Xiamen University, which included 26 (62.5%) females and 14 (37.5%) male cases aged 48 years (range, 36–75) on average. As expected, all of the three diagnostic markers showed statistical significance (*p* < 0.05) and were down-regulated in the SONFH group in comparison with the non-SONFH group (Figures 6C–E).

### Infiltration of Immune Cells Results

**FIGURE 7:**
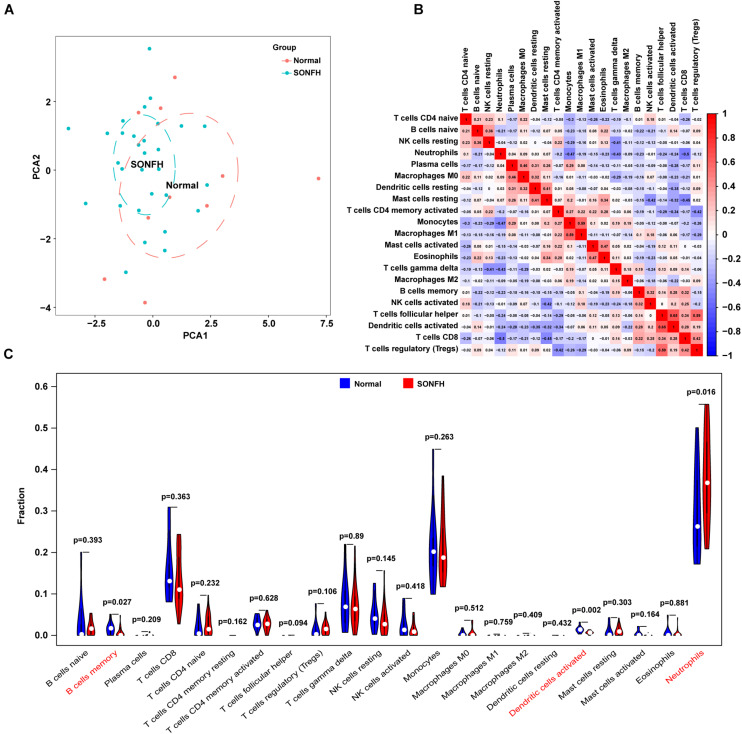
Evaluation and visualization of immune cell infiltration. **(A)** PCA cluster plot of immune cell infiltration between SONFH samples and control samples. **(B)** Correlation heat map of 22 types of immune cells and one kind of immune cell with no difference were removed. The size of the colored squares represents the strength of the correlation; red represents a positive correlation and blue represents a negative correlation. **(C)** Violin diagram of the proportion of 22 types of immune cells. The red marks represent the difference in infiltration between the two groups of samples.

With CIBERSORT software, the infiltration status of 22 types of immune cells between SONFH and normal cases were assessed. A significant difference was identified in the infiltration of immune cells between the two groups from PCA cluster analysis results (Figure 7A). After conducting a correlation analysis of infiltrated immune cells, we found multiple pairs of positively and negatively related immune cells (Figure 7B). The degree of correlation was represented by the score. This result suggests that CD8 T cells and neutrophils, CD8 T cells and resting mast cells, and monocytes and neutrophils had a significant negative correlation, respectively. Activated dendritic cells and follicular helper T cells, regulatory T cells, and follicular helper T cells, and M1 macrophages and monocytes had a significant positive correlation, respectively. As indicated from the violin plot, compared with the normal control sample, activated dendritic cells and memory B cells infiltrated less, while neutrophils infiltrated more often (Figure 7C).

### Correlation Analysis Between Diagnostic Markers and Infiltration-Related Immune Cells

**FIGURE 8:**
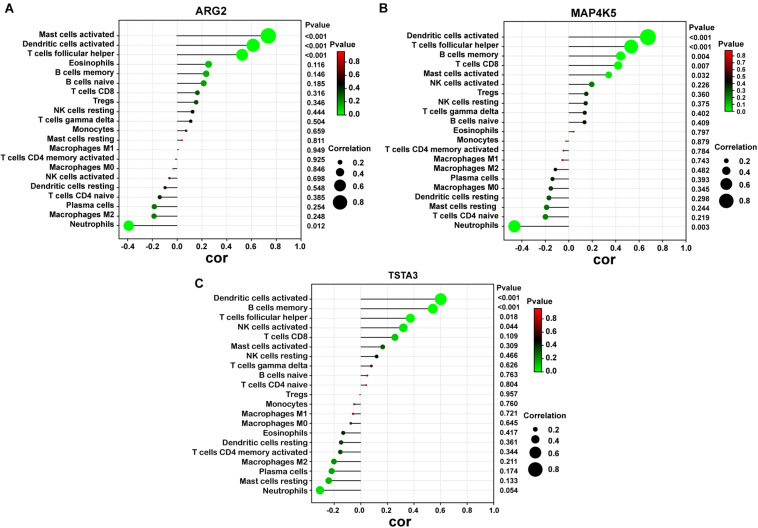
Correlation between diagnostic markers and infiltrating immune cells. **(A)** Correlation between ARG2 and infiltrating immune cells. **(B)** Correlation between MAP4K5 and infiltrating immune cells. **(C)** Correlation between TSTA3 and infiltrating immune cells. The size of the dots represents the strength of the correlation between genes and immune cells; the larger the dots, the stronger the correlation, and the smaller the dots, the weaker the correlation. The color of the dots represents the *p*-value, the greener the color, the lower the *P*-value, and the red the color, the larger the *p*-value. *P* < 0.05 was considered statistically significant.

As indicated from the correlation analysis, ARG2 displayed a positive correlation with activated mast cells (*r* = 0.762, *p* < 0.001), activated dendritic cells (*r* = 0.612, *p* < 0.001) and T cells follicular helper (*r* = 0.536, *p* < 0.001) and a negative correlation with neutrophils (*r* = –0.408, *p* = 0.012). MAP4K5 displayed a positive correlation with activated dendritic cells (*r* = 0.682, *p* < 0.001), T cells follicular helper (*r* = 0.576, *p* < 0.001), and B cells memory (*r* = 0.439, *p* = 0.004) and a negative correlation with neutrophils (*r* = −0.438, *p* = 0.003). TSTA3 positively correlated with activated dendritic cells (*r* = 0.607, *p* < 0.001), B cells memory (*r* = 0.579, *p* < 0.001), as well as T cells follicular helper (*r* = 0.368, *p* = 0.018) (Figure 8).

## Discussion

SONFH is a progressively crippling disease of the femoral head, of which the pathogenesis is complex and multifactorial, with environmental and genetic factors exerting vital effects (Chang et al., 2020). Clinically, for the lack of typical symptoms and sensitive diagnostic indicators, it is sometimes difficult to predict and diagnose cases with SONFH and thus lose the optimal treatment opportunity. Moreover, as indicated from existing studies, immune cells primarily impact the development of SONFH (Ma et al., 2019). For this reason, there is profound significance in finding specific diagnostic markers and analyzing the pattern of infiltration of immune cells for improving the prognosis of SONFH cases. In this study, we sought to screen and determine the diagnostic markers of SONFH and further explore the relationship between infiltration-related immune cells and diagnostic markers.

On the whole, 383 DEGs were identified. As demonstrated from GO enrichment results, DEGs showed a major relationship to neutrophil activation, neutrophil mediated immunity, neutrophil degranulation, and the neutrophil activation involved in the immune response. To be specific, the immune system was linked remarkably with SONFH occurrence and development from the results of GO analysis. Neutrophils derived from bone marrow and acting as the vital components of the body’s natural immunity were suggested to have an important functional role in the body’s defense against invasive factors (Jablonska et al., 2017). Jiang et al. (2019) reported that the higher the percentage of neutrophil cells, the more likely patients were to get femoral head necrosis. The findings of existing studies are consistent with those drawn in this study using different methods. Wu’s team selected the hub genes in the key module using WGCNA and then performed GO analysis. They found the top 10 hub genes involved in the inflammatory response (Wu et al., 2021). Furthermore, the pathway enriched by GSEA was mainly involved in osteoclast differentiation and rheumatoid arthritis. It has been extensively reported that there were several imbalances in femoral head necrosis (e.g., osteoblast apoptosis and differentiation, bone resorption and formation), thereby causing necrosis around the subchondral bone of the femoral head (Björkman et al., 2004; Lau et al., 2014). The study suggested that by performing animal experiments that glucocorticoids cause disorder of osteoclast differentiation by down-regulating the expressions of β-catenin and c-Myc downstream of the Wnt pathway, resulting in early SONFH (Zhang et al., 2015). As is generally known, rheumatoid arthritis is a chronic autoimmune disease. The pathway of rheumatoid arthritis might also indicate the development of SONFH was related to inflammation. The above research reflects the results of the functional enrichment analyses of the present study, indicating the accuracy of our results.

LASSO logistic regression, a machine learning algorithm, determined the variable by searching for λ under the smallest probability of classification error (Suykens and Vandewalle, 1999). SVM-RFE is a machine learning algorithm that complies with statistical learning theory to search the terms of the optimal variable through the subtraction of the feature vector developed (Cherkassky, 1997). Random forests (RF) consisted of an ensemble decision tree in which each internal node corresponds to a test on an attribute for classification (Breiman, 2001). WGCNA is capable of integrating gene expressing state and trait information in an effective manner for the identification of function-related channels and candidate biological markers (Presson et al., 2008). We integrated the four different algorithms, each of which has inherent characteristics. Finally, ARG2, MAP4K5, and TSTA3 were selected and were accurate according to further validations in this study, which suggested that our prediction was feasible by the integration strategy. Previous studies have identified diagnostic markers using the GSE123568 datasets via different methods. Wu et al. (2021) picked *RHAG, RNF14, HEMGN*, and *SLC2A1* as the key genes for SONFH by integrating WGCNA analysis and the DEGs with the top degree of connectivity (Wu et al., 2021). In order to reduce the number of key genes (choosing 91 hub genes form the brown module with 1258 genes), Wu et al. (2021) set an overcritical cut-off value, which might indicate the possibility that some important candidate genes were missed. The results of Shile et al. (2021) were different from our results due to them using different algorithms (Shile et al., 2021). However, ARG2 was also identified as a key gene for SONFH through ingenuity pathway analysis, which also indicates that our prediction was feasible by the integration strategy. Three diagnostic genes were down-regulated and ranked in the top 60 based on adjusted *P*-values among a total of 383 DEGs. Although several previous studies also identified gene signatures by incorporating all DEGs, the results are not entirely consistent with each other. The main reason for the differences is that different values were applied as cut-off thresholds. Wu et al. (2021) selected | log_2_(fold change)| > 1.5, while Shile et al. (2021) set with a threshold false discovery rate < 0.25. These factors are bound to result in differences in conclusion. The two studies lacked a further trial to validate the results, so we argue that our results are more comprehensive and accurate.

There were two arginase isoforms, including ARG2, which expressed predominantly in the extra-hepatic. This study demonstrated that ARG2 played a role in the modulation of polyamine metabolism and nitric oxide by regulating local arginine concentrations (Vockley et al., 1996). As reported by earlier studies, the content of nitric oxide is positively linked to the index of osteocyte apoptosis, indicating that nitric oxide induced osteocyte apoptosis (Bai et al., 2015). Coincidentally, according to several studies, abnormal arginase expression displayed significant correlations with multiple pathological processes, such as ischemic stroke (Lyu et al., 2020), cardiovascular (Berkowitz et al., 2003), and immune-mediated (Morris, 2016). Clemente et al. (2020) also reported that arginase was considered a potential biological marker of disease severity and progression and also acted as the research subject based on the therapeutic efficacy of arginase inhibitors.

The Ste20-like family of kinase was a complex signaling cascade that consisted of a wide range of kinase mediators, including MAP kinase kinases (MAP4Ks), one of which was called MAP4K5. MAP4K5 is also named germinal center kinase related (GCKR) or kinase homologous to STE20 (KHS1), phosphorylating multiple downstream kinase cascades (Strange et al., 2006; Vin et al., 2013). As reported by Shi et al. (1999), MAP4K5 regulated JNK activating process within TNFα signaling and might impact the inflammation responses under the relationship to TNFα. MAP4K5 was also involved in CD40 signaling (Chen et al., 1999), which played important functions in the memory B-cell forming process, germinal center, and DC maturation and activating process.

The TSTA3, i.e., GDP-fucose synthase (FX), was an NADPH-binding protein and critically impacted glycosylation (Tonetti et al., 1996). Glycosylation participates in some important biological processes, with vital effects on the regulation of the function of proteins and cells, as well as various biological functions (e.g., cell signaling, cell immunogenicity, cell–cell and cell–substrate interactions, adhesion) (Moremen et al., 2012; Varki, 2017). Research evidenced that the identified diagnostic markers may be signally connected with the occurrence and progression of SONFH.

The present study adopted CIBERSORT to evaluate the role of the infiltration of immune cells in SONFH. According to the results of this study, an improved infiltration of neutrophils, and an inhibited activated dendritic cell infiltration and memory B cells might be related to SONFH occurrence and development. It has been shown that the percentage of neutrophils had a significant association with a greater femoral head necrosis incidence (Jiang et al., 2019). Poubelle et al. (2007) found that neutrophils participated in bone remodeling processes and local immune responses. Previous studies have shown that the imbalance in bone formation and bone resorption was the important cause of SONFH (Shi et al., 2007). Neutrophils might inhibit the biological processes of the osteoclast forming process and accelerate bone resorption, resulting in femoral head necrosis. B cells are mainly responsible for humoral responses. When an antigen stimulated a B cell, the B cell produces considerable memory B cells or antibody-secreting plasma B cells. Prior studies have suggested that the contents of circulating CD19^+^ cells [a signal transduction molecule of B cells in both memory and naïve B cells (Sato et al., 2000)] were positively related to the index of femoral head collapse in SONFH (Zhang et al., 2014). According to previous studies combined with the analysis results achieved by this study, neutrophils and memory B cells critically impacted SONFH and should receive major in-depth research. Nevertheless, no relevant research on the roles of activated dendritic cells in SONFH has been conducted to date. According to existing research, the combination with the analysis results achieved by this study, activated NK cells, resting CD4 memory T cells, regulatory T cells, and resting mast cells critically impact SONFH and are required to be primarily studied in depth. Yet the effect exerted by eosinophils in SONFH has not been studied, and in-depth experiment-related information should be presented. Furthermore, the results of this study reveal specific information on infiltration immune cells in SONFH. CD8 T cells and neutrophils, resting mast cells and CD8 T cells, and monocytes and neutrophils displayed a noticeable negative correlation, respectively. Dendritic cells under the activation and follicular helper T cells, follicular helper T cells, and regulatory T cells. In addition, Monocytes and M1 Macrophages displayed a remarkable positive relationship, respectively.

As suggested from the relationship of diagnosis-related markers and infiltration-based immune cells, ARG2 displayed a significant association with activated mast cells, activated dendritic cells, T cells follicular helper, and neutrophils. MAP4K5 was significantly associated with activated dendritic cells, T cells follicular helper, B cells memory, and neutrophils. Moreover, TSTA3 was significantly correlated with activated dendritic cells, B cell memory, as well as T cells follicular helper. As indicated by existing research, polymorphonuclear neutrophils and mast cells could produce or activate destructive enzymes, thereby causing cartilage destruction and even bone necrosis (Mohr, 1996). The absence of dendritic cells could lead to osteonecrosis following dental extraction in experimental animals (Elsayed et al., 2020). Existing studies have demonstrated that glucocorticoid contributed to local ischemia of the femoral head by inducing activation of platelets (Lang et al., 2015), thereby causing neutrophil extracellular traps (Nonokawa et al., 2020). Given all the information acquired by the authors, this study initially reported the prediction of the correlations of immune infiltration in SONFH, and the complex interactions require further research and clarification.

In this study, novel scientific approaches were adopted to identify SONFH diagnostic markers (e.g., RF algorithms, WGCNA, LASSO logistic regression, and SVM-RFE). Moreover, this study initially used CIBERSORT for assessing the infiltration of immune cells between normal tissues and SONFH tissues. However, this study had certain limitations and deficiencies. First, though the analysis results achieved were compatible with some previous research results, the sample size was insufficient to validate the study. The results thus should be validated by a large sample study. Second, CIBERSORT investigation complied with the genetic information of a limited amount but this data is not likely to show the deviation from phenotypic plasticity, disease-induced disorders, or the heterotypic interactions of cells. Third, our study performed the second mining and investigation of existing datasets. Additionally, age was usually treated as a categorical variable in all clinical studies, but information relating to age could not be extracted. There might also be limitations in terms of biased results and conclusions, therefore further relevant studies are warranted.

## Conclusion

The present study reported that ARG2, MAP4K5, and TSTA3 were potential diagnosis-related genes in SONFH. In addition, neutrophil activated dendritic cells and memory B cells may be related to SONFH occurrence and progress. Moreover, ARG2 displayed a noticeable association with activated mast cells, activated dendritic cells, T cells follicular helper, as well as neutrophils. MAP4K5 was significantly associated with T cells follicular helper, activated dendritic cells, B cells memory, and neutrophils. TSTA3 was significantly associated with T cell follicular helper, B cell memory, and activated dendritic cells. These immune cells may serve an important role in the development of SONFH. These immune cells may facilitate in-depth exploration of targeted immunotherapeutic methods and optimize immunomodulation therapies for cases of SONFH.

## Data Availability Statement

Table 1 showed the baseline characteristics of the study population. The original contributions presented in the study are included in the article/Supplementary Material, further inquiries can be directed to the corresponding author/s.

**TABLE 1:** Baseline characteristics of the study population.

Characters	Overall	SONFH (*n* = 24)	non-SONFH (*n* = 16)	*P*
Female (%)	26 (62.5%)	16 (61.5%)	10 (38.5%)	0.073
Male (%)	14 (37.5%)	8 (57.1%)	6 (42.9%)	
Age (year)	48 (36–75)	42 (41–75)	46 (36–71)	0.682
MBI (kg/m^2^)	24.7 ± 2.3	24.6 ± 2.4	25.1 ± 1.4	0.321
ARCO I–II (%)	8 (33.3%)	8 (33.3%)	0	
ARCO III (%)	8 (33.3%)	8 (33.3%)	0	NA
ARCO IV (%)	8 (33.3%)	8 (33.3%)	0	

*ARCO, Association Research Circulation Osseous; SONFH, steroid-induced osteonecrosis of the femoral head; NA, Not applicable; MBI, Body Mass Index.*

## Ethics Statement

The study was approved by the Clinical Research Ethics Committee of Fuzhou Second Hospital Affiliated to Xiamen University. Each patient provided written signed informed consent (Project identification code: 2021001). The patients/participants provided their written informed consent to participate in this study.

## Author Contributions

YYZ and RY: conception and design of the study and funding acquisition. RY, JZ, and YGZ: data acquisition, bioinformatics analysis, and drafting and critical revision of the manuscript. RY, XH, ST, JY, and NL: visualization and validation. All authors approved the final manuscript.

## Conflict of Interest

The authors declare that the research was conducted in the absence of any commercial or financial relationships that could be construed as a potential conflict of interest.

## Publisher’s Note

All claims expressed in this article are solely those of the authors and do not necessarily represent those of their affiliated organizations, or those of the publisher, the editors and the reviewers. Any product that may be evaluated in this article, or claim that may be made by its manufacturer, is not guaranteed or endorsed by the publisher.

## Footnotes

1 http://www.ncbi.nlm.nih.gov/geo/

2 https://www.ncbi.nlm.nih.gov/geo/query/acc.cgi?acc=GSE123568

## Supplementary Material

Supplementary List 1 The list of 119 up-regulated and 264 down-regulated genes.

## Abbreviations

SONFH: Steroid-induced osteonecrosis of the femoral head
DEGs: Differentially expressed genes
ARCO: Association Research Circulation Osseous
PCA: Principal component analysis
SVM-RFE: Support vector machine-recursive feature elimination
WGCNA: Weighted gene co-expression network analysis
LASSO: Least absolute shrinkage and selection operator
RF: Random forests
qRT-PCR: Quantitative reverse transcriptase PCR
ROC: Receiver operating characteristic
AUC: Area under the curve
SARS: Severe acute respiratory syndrome
GEO: Gene Expression Omnibus
RMA: Robust multiarray averaging
GO: Gene Ontology
KEGG: Kyoto Encyclopedia of Genes and Genomes
GSEA: Gene set enrichment analysis
CT: Cycle threshold
GCKR: Germinal center kinase related
FX: Fucose synthase.
